# Editorial: Post-traumatic Stress Disorder and Complex Traumatic Stress Disorder in Children and Adolescents

**DOI:** 10.3389/fpsyt.2021.661488

**Published:** 2021-04-01

**Authors:** Jonathan Lachal, Mayssa' El Husseini, Cécile Rousseau, Marie Rose Moro

**Affiliations:** ^1^CHU de Clermont-Ferrand, Service de Psychiatrie de l'Enfant et de l'Adolescent, Clermont-Ferrand, France; ^2^Université Clermont Auvergne, Clermont-Ferrand, France; ^3^Université Paris-Saclay, UVSQ, Inserm, CESP, Team DevPsy, Villejuif, France; ^4^Université Picardie Jules Verne, Centre d'Histoire des Sociétés, des Sciences et des Conflits, EA 4289, Amiens, France; ^5^Division of Social and Transcultural Psychiatry, McGill University, Montreal, QC, Canada; ^6^Hôpital Cochin, Maison de Solenn, AP-HP, Paris, France; ^7^Université de Paris, PCPP, Boulogne-Billancourt, France

**Keywords:** cross cultural approach, PTSD, CPTSD, collective trauma, trauma transmission

Psychological trauma constitutes a determinant experience of adversity in the emotional development of children and adolescents. Unidentified and untreated traumatic experiences, sometimes cumulative, can interfere with the child's development and impair his or her psychological and somatic functioning, leading in some cases to the development of post-traumatic stress disorder (PTSD) or complex post-traumatic stress disorder (CPTSD).

*Post-Traumatic Stress Disorder and Complex Traumatic Stress Disorder in Children and Adolescents* explores the many facets of traumatic experiences encountered in childhood and adolescence. Authors from several professional and cultural backgrounds present clinical and original research work in varied settings. They are especially mindful of the cross-fertilization of evidence-based and culture-relevant therapeutic techniques. In these studies, the patients come from all over the world, from Asia, Africa, the Middle East, the Americas, and Europe. Some of the studies also deal with specific migrant populations who are susceptible to PTSD and CPTSD. Particular attention is also paid to transcultural factors and their impact on symptoms and treatment.

The authors, clinical practitioners and researchers, opted for an open definition of traumatic disorders, broader than the strict psychiatric definition of PTSD. Their clinical practice shows a vast array of traumatic symptoms triggered by external distressing events. They also sheds light on traumatic symptoms induced by internal conflicts related to the important changes in the body during puberty and adolescence. During infancy, deficiencies in early interactions between babies and their caretakers can obstruct the emotional regulation process.

Trauma is a psychophysical experience, and traumatized patients suffer from diverse somatic symptoms (1, 2). However, little is known about the relation between sleep disorders, depression, and PTSD in refugee children. Park et al. show that depression may mediate the links between PTSD and insomnia. Tarazi-Sahab et al. discuss the potentially traumatogenic role puberty can play in adolescence. Verelst et al. consider an *a priori* pathological behavior—avoidant/disengagement coping—as a protective factor against post-traumatic stress and anxiety.

The environments of children and adolescents play an important role in the evolution of their direct or indirect exposure to a highly distressing event into a stress-related disorder. The family, social, and cultural environments can either acknowledge the experience and support these youth, or they can deny it and thus exacerbate the problems. El-Khodary et al. and Brown et al. study these complex interrelationships in two quite different contexts: the first among Palestinian children living in the Gaza Strip, essentially a war zone, and the second among students after an ecological disaster (a wildfire).

Two studies by Grenon et al. and Gindt et al. look at collective violence, presenting study protocols on recent terrorist attacks in Europe. There is a serious lack of data about the direct and long-term consequences of exposure to terrorist attacks and mass murders in childhood and adolescence. Both studies provide valuable contributions to our understanding of the impact of collective traumatic events intentionally inflicted by other humans.

Qualitative approaches, including narrative, are valuable in increasing our understanding of the relations between traumatic event, child-parent interaction, and post-traumatic symptoms. Radjack et al. developed a research-action program aiming to transmit cultural know-how to social workers who provide care for migrant youths traveling without their parents. Based on a transcultural approach, the program aims to assist these migrant youths in developing, through narrative, their bicultural adolescences. Klein et al. tackle the complex issue of how to deal with underage children, born or taken by their parents to jihadist group operation areas, when they return to France. In both articles, the authors resort to structuring narratives in their approaches. Finally, Roques et al. present a protocol where interviews and quantitative data will be collected from psychological tests to explore the complex links between bullying and PTSD in adolescence.

For children, trauma may also be experienced indirectly through transgenerational transmission of trauma from parents (or other primary caregivers) to children (3). The mechanisms behind this transmission of parental trauma in complex settings such as war or migration nonetheless remain unclear. The study by Dozio et al. on this topic shows how the mother, in narrating her trauma, can disconnect from or detune her interaction with her infant and what repercussions it can have on the infant's behavior and interaction.

In some cases, unidentified and cryptic traumatic experiences may manifest through symptoms and relational dysfunctions in situations where a part of the child's history is unknown. Mansouri et al. mixed sociological, psychological, and transcultural approaches to broaden their perspective on the French “riots” of 2005. Their model includes the traumatic impact of past violence in France's colonial history. The authors argue that the collective silencing of the colonial past, by preventing the necessary discussion/narration of past traumatic experiences, contributes to the acting out of violence that lacks other means of expression.

Less is known about ascendant transmission of trauma. When babies and children are exposed to traumatic experiences, what impact does this exposure to trauma have on caregivers' representations and care abilities? Skandrani et al. explored this issue in adoption contexts. They highlighted the need for a support program for parents in their adoption procedure, to enable better parental preparation to welcome a child and help the child alleviate the trauma's impact on his or her emotional development. Nascimento et al. studied the representations that social caregivers working in childcare shelters have of the babies' lives before institutionalization. The psychological impact of these representations is frequently underestimated, and building narratives where children overcome the marks and frustrations caused by abandonment in early childhood and living in an institution appears an effective and potentially important way of preventing emotional distress.

Narrative approaches are preferred by many authors for therapy for PTSD and CPTSD. Narrative Exposure Therapy is a well-known method developed for adult PTSD (4), but few studies have examined its use with children, and even fewer for childhood CPTSD. Fazel et al. describe its adaptation for children and adolescents in different contexts, with promising results. In a different type of narrative strategy, El-Khodary et al. present a complete school-based narrative intervention with students to prevent the emergence of PTSD symptoms after exposure to war related trauma.

The original research articles and clinical cases in *Post-Traumatic Stress Disorder and Complex Traumatic Stress Disorder in Children and Adolescents* address the multiple configurations of events leading to the development of traumatic symptoms or PTSD: collective and individual traumatic experiences, event-related and internal conflict-related traumatic constellations. A qualitative approach to PTSD and CPTSD in children and adolescents opens the way to an in-depth discussion about adjusting therapeutic strategies to the particular traumatic experience and the available resources.

## Author Contributions

JL, ME, CR, and MM wrote the manuscript and gave final approval. All authors contributed to the article and approved the submitted version.

## Conflict of Interest

The authors declare that the research was conducted in the absence of any commercial or financial relationships that could be construed as a potential conflict of interest.

## References

[1] Van der Kolk BA Pelcovitz D Roth S Mandel FS McFarlane A Herman JL. Dissociation, somatization, and affect dysregulation: the complexity of adaptation of trauma. Am J Psychiatry. (1996) 153:83–93. 10.1176/ajp.153.7.83 8659645

[2] Ogden P Minton K Pain C. Trauma and the Body – A Sensorimotor Approach to Psychotherapy. New York: Norton Professional Books (2006).

[3] Ancharoff M Munroe J Fisher L. The legacy of combat trauma: clinical implications of intergenerational transmission. In: International Handbook of Multigenerational Legacies of Trauma. New York, NY: Plenum Press (1998). p. 257–76. 10.1007/978-1-4757-5567-1_17

[4] Robjant K Fazel M. The emerging evidence for narrative exposure therapy: a review. Clin Psychol Rev. (2010) 30:1030–9. 10.1016/j.cpr.2010.07.004 20832922

